# Facile encapsulation of cyanoacrylate-based bioadhesive by electrospray method and investigation of the process parameters

**DOI:** 10.1038/s41598-024-56008-2

**Published:** 2024-03-05

**Authors:** Alireza Aminoroaya, Saied Nouri Khorasani, Rouholah Bagheri, Zahra Talebi, Roya Malekkhouyan, Oisik Das, Rasoul Esmaeely Neisiany

**Affiliations:** 1 Department of Chemical Engineering, Isfahan University of Technology, Isfahan, 84156-83111 Iran; 2 Department of Chemical Engineering and Materials Science, Michigan State University, 428 S. Shaw Lane, East Lansing, MI 48824 USA; 3 Department of Textile Engineering, Isfahan University of Technology, Isfahan, 84156-83111 Iran; 4 Department of Civil, Environmental and Natural Resources Engineering, Luleå University of Technology, 97187 Lulea, Sweden; 5 Biotechnology Centre, Silesian University of Technology, Krzywoustego 8, 44-100 Gliwice, Poland; 6 Department of Polymer Engineering, Hakim Sabzevari University, Sabzevar, 9617976487 Iran

**Keywords:** Encapsulation, Bioadhesive, Microcapsules, Electrospray, Taguchi design, Engineering, Materials science

## Abstract

Polymer microcapsules containing cyanoacrylates have represented a promising option to develop self-healing biomaterials. This study aims to develop an electrospray method for the preparation of capsules using poly(methyl methacrylate) (PMMA) as the encapsulant and ethyl 2-cyanoacrylate (EC) as the encapsulate. It also aims to study the effect of the electrospray process parameters on the size and morphology of the capsules. The capsules were characterized using Fourier-transform infrared (FTIR) spectroscopy, thermogravimetric analysis (TGA), and field-emission scanning electron microscopy (FE-SEM). Moreover, the effects of electrospray process parameters on the size were investigated by Taguchi experimental design. FTIR and TGA approved the presence of both PMMA and EC without further reaction. FE-SEM micrograph demonstrated that an appropriate choice of solvents, utilizing an appropriate PMMA:EC ratio and sufficient PMMA concentration are critical factors to produce capsules dominantly with an intact and spherical morphology. Utilizing various flow rates (0.3–0.5 ml/h) and applied voltage (18–26 kV), capsules were obtained with a 600–1000 nm size range. At constantly applied voltages, the increase in flow rate increased the capsule size up to 40% (ANOVA, *p* ≤ 0.05), while at constant flow rates, the increase in applied voltage reduced the average capsule size by 3.4–26% (ANOVA, *p* ≤ 0.05). The results from the Taguchi design represented the significance of solution flow rate, applied voltage, and solution concentration. It was shown that the most effective parameter on the size of capsules is flow rate. This research demonstrated that electrospray can be utilized as a convenient method for the preparation of sub-micron PMMA capsules containing EC. Furthermore, the morphology of the capsules is dominated by solvents, PMMA concentration, and PMMA:EC ratio, while the average size of the capsules can be altered by adjusting the flow rate and applied voltage of the electrospray process.

## Introduction

Encapsulation of highly active materials prolongs the lifespan and provides effective protection which has been the most common method for protecting, controlling the release process, and enhancing the quality of active materials^1–3^. Therefore, enclosing the desired material (encapsulate) in the specific encapsulant can protect it against degradation and deterioration^4–6^. Encapsulating has been used for different applications, including the food industry^7^, drug delivery^8,9^, diagnosis and treatment^10^, and self-healing approaches in biomedical materials, coatings, and polymer matrix composites^11–14^. Encapsulation of cyanoacrylate bioadhesives has been investigated for the development of self-healing bone cement, but their encapsulation was highly challenging^15,16^. These cyanoacrylate monomers are highly moisture reactive, which can trigger their polymerization during the encapsulation process^17^. Encapsulating moisture-reactive materials using conventional emulsion-based methods necessitates the use of various chemical inhibitors to avert premature polymerization. However, this approach can compromise the performance of cyanoacrylate bioadhesives upon application and adversely impact their biocompatibility. Consequently, there is a pressing need to develop an alternative encapsulation technique for cyanoacrylate bioadhesives that effectively overcomes the aforementioned challenges.

Different methods of encapsulation have been developed and reported in the literature, including interfacial polymerization, in situ polymerization, solvent evaporation, multi-orifice centrifugal process, air suspension, electrospinning, and electrospray^18–20^. Choosing the encapsulation process can highly affect the properties of capsules, such as encapsulation efficiency, the content of the encapsulated materials, size distribution, and mean diameter^21–23^. Among these methods, electrospray has gained significant attention due to its ability to encapsulate a wide range of materials with high encapsulation efficiency, and minimum use of chemicals i.e. initiators, and emulsifiers^24–26^. These features suggest that electrospray is an excellent method for encapsulating reactive cyanoacrylate bioadhesives. The electrospray process can be performed using a high-voltage supply providing a sufficiently strong electrical force for converting the outlet droplets to smaller particles^27,28^. This is due to the Rayleigh effect, which occurs when the amount of charge on droplets overcomes their surface tension. These smaller droplets accelerate in the electrical field and lose their solvent (evaporation of solvent). It causes an increase in charge density and Coulombic eruption leading to even smaller particles^29^. As expressed in the literature, effective electrospray parameters consist of solution flow rate, applied voltage, needle-to-collector distance, solution concentration, and solvent types^27,30^. There are various modes of electrospray from dripping mode to different jet modes. These modes form depending on the selected amounts for electrospray parameters. The desired mode is the cone jet geometry because of its stability and production of particles with appropriate morphology^31,32^.

Within stable electrospray process conditions, the size of produced particles is influenced by properties of the precursor feed, including molecular weight of encapsulate and encapsulants, their concentrations, and solvent(s) types, as well as process-related parameters, mainly flow rate and applied voltage^31,33^. For such a multi-variable process, utilization of a design of experiment method is of great importance in order to not only study the effect of each parameter on the size of the capsules individually but also to determine the simultaneous effect of the parameters on the capsule size. In this regard, the Taguchi method is a frequently used approach that has been utilized to study the effect of electrospray parameters on the characteristics of produced capsules^34,35^. This orthogonal array design method, which was originally introduced by Genichi Taguchi, enables investigating multiple parameters with a minimum number of required experiments^36,37^. This method can be used for optimizing a process with respect to a goal (e.g. achieving the lowest capsule size) including the determination of the level of significance and process variability for each process parameter. In a previous encapsulation study by electrospray, M. K. Moghadam et al. utilized the Taguchi method for optimizing the alginate encapsulation of n-nonadecane by alginate using a coaxial electrospray method. By investigating the electrospray process parameters, feed properties, and needle geometry, the authors determined that the optimal electrospray parameters resulted in n-nonadecane/alginate capsules with a size range of 80–350 nm^35^. In another extensive study, J Roine et al. utilized the Taguchi method to study ten electrospray process parameters on the process efficiency of capsules with Eudragit E 100 copolymer as shell and sodium iodide-doped glycerol containing carbonized porous silicon as core, using a parallel nozzle electrospray method. According to the unique feature of the electrospray encapsulation process, its potential application for the encapsulation of cyanoacrylate bioadhesives seems to be promising.

Therefore, in this study, the capsules with PMMA as encapsulant and EC as encapsulate are prepared by the single-nozzle electrospray method. Moreover, the characterization of capsules was carried out using FTIR, FE-SEM, and TGA. Taguchi method was also adopted to investigate the effect of electrospray parameters, including precursor formulation, flow rate, and applied voltage, on the size of capsules, and the process parameters were optimized to yield capsules with the lowest size. This is the first study regarding the encapsulation of a cyanoacrylate bioadhesive by electrospray method which represents a facile method for the fabrication of such micron-sized for biomedical applications.

## Materials and methods

### Materials

Poly (methyl methacrylate) (PMMA, MW = 120,000), *N*, *N*-dimethylformamide (DMF, anhydrous 99.8%, product no. 227056), and dichloromethane (DCM, ≥ 99.9%, product no. 650436) were provided by Sigma-Aldrich (St. Louis, MO, USA). Ethyl 2-cyanoacrylate (EC, Epiglue) was purchased from Meyer-Haake (Germany). Chemicals were utilized as received without any extra purification.

### Solution preparation

Six solutions containing PMMA and EC were prepared for the electrospray process according to the composition listed in Table 1. Briefly, PMMA was dissolved in 10 ml of solvent, which was DMF, DCM, or 1:1 (v/v) DMF:DCM, by magnetic stirring in a sealed glass bottle at ambient conditions (room temperature of 20–25 °C, atmospheric pressure, and relative humidity of 20–30%) for 12 h to prepare. Afterward, the EC was added precisely to the solution, and magnetic stirring was maintained for 2 h to obtain a homogeneous solution. All of the steps for solution preparation were carefully conducted in completely dry conditions to avoid pre-mature polymerization of EC. Once the PMMA:EC solution was prepared, it was used for the electrospray process.

**Table 1:** The composition of various solutions utilized for the electrospray process to prepare PMMA/EC capsules.

Solution code	Solvent(s)	PMMA% (g/ml)	PMMA:EC (wt/wt)
S_1_	DMF:DCM (1:1 v/v)	2.5	50:50
S_2_	DMF:DCM (1:1 v/v)	3	60:40
S_3_	DMF:DCM (1:1 v/v)	4	60:40
S_4_	DMF	1.5	60:40
S_5_	DCM	2	60:40
S_6_	DCM	2.5	60:40

### Capsule preparation

**Figure 1:**
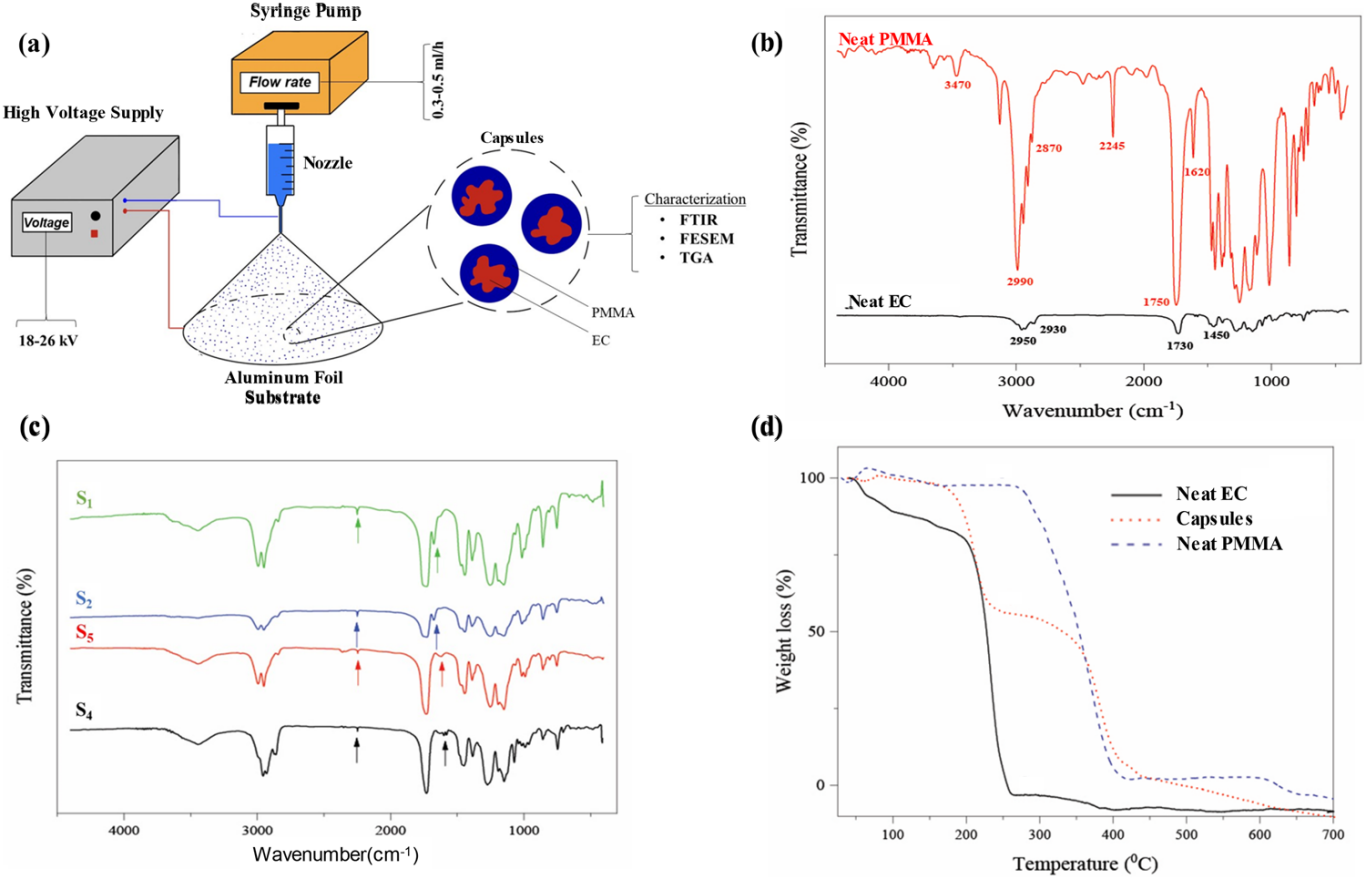
Preparation, chemical composition, and thermogravimetry of PMMA/EC capsules: (**a**) schematic representation of the electrospray process utilized for the preparation of capsules and following characterizations. The components include a high voltage supply, syringe pump, syringe and nozzle assembly, aluminum foil substrate, and schematic magnification of prepared capsules. (**b**) FTIR spectra of neat PMMA, EC (n = 3), and (**c**) FTIR spectra of capsules prepared by electrospray of S_4_, S_2_, S_4_, and S_5_ solutions (n = 3) reveal the presence of EC in the PMMA capsules without any pre-mature reaction. (**d**) The TGA curves of neat EC, prepared capsule, and neat PMMA further represent the presence of the two components, EC and PMMA in the capsule structure (n = 3).

The solutions containing PMMA and EC, as described in “Solution preparation” section, were prepared for the electrospray process^33,38^. In the following, solutions were applied to a 10 ml glass syringe with a blunt steel needle, gauge 23 (Klarglas, Germany). After applying anode and cathode to the collector (10 × 10 cm^2^ aluminum foil) and needle, respectively, electrospray was carried out with different voltages from 18 to 26 kV, and flow rates from 0.3 to 0.5 ml/h as specified in Table 2. The needle tip-to-collector distance was set at 20 cm for all electrospray processes. The configuration of the electrospray process is depicted in Fig. 1a. Throughout each electrospray run, the process was monitored for the presence of a stable cone jet geometry and overall uniform particle production^33^. The cone jet geometry was monitored visually, and overall uniform particle production was monitored by regularly sampling particles using a glass microscope slide and observing them under an optical microscope (HP31, China) with $\times$ 100 magnification. Capsules collected at desired electrospray process conditions were stored at − 18 °C and further used for FTIR, FESEM, and TGA analysis.

**Table 2:** Three effective factors and their range were investigated to prepare capsules using each solution.

Factors	Unit	Symbol	Levels		
PMMA/EC concentration ratio		C	1	2	
Voltage	kV	V	18	22	26
Flow rate	ml/h	I	0.3	0.4	0.5

### Capsule characterization

For investigating the chemical structure of the neat materials and prepared capsules FTIR spectrometer (WQF-510A, China), with 32 scans in the wavenumber range of 4000 to 500 cm^−1^ at the resolution of 4 cm^−1^, was used. The morphology, size, and shape of the fabricated capsules were assessed by FE-SEM (QUANTA FEG 450, Graz, Austria). Finally, the thermal stability of prepared capsules was evaluated using a TGA (Perkin Elmer STA 6000 TGA system, USA). The TGA tests were conducted, under an N_2_ atmosphere, at the heating rate of 10 °C min^−1^ with a temperature range of 25 to 700 °C. Figure 1 represents the process of preparation and characterization results of the capsules schematically.

### Experimental design

Taguchi method was used to reveal the effects of electrospray parameters on the encapsulation process^39^. This method uses the Signal to Noise ratio (S/N) to investigate the effectiveness of levels of electrospray parameters on the final result. The S/N ratio measures the relation of desired responses or results to the standard deviation or noise^40^. Moreover, the significance of parameters was statistically validated by analysis of variance (ANOVA). Therefore, the experiments were designed to reveal how the size of capsules is influenced by the electrospray parameters, including the PMMA: EC concentration ratio, the flow rate, and applied voltage. The flow rate was considered at three levels of 0.3, 0.4, and 0.5 ml/h, and applied voltage was considered at three levels of 18, 22, and 26 kV. PMMA: EC concentration ratios were considered at two levels of 1 and 2 which stands for 50:50 and 60:40 (wt/wt), respectively. It is worth mentioning that both states have a cumulative concentration of 5% (g/ml), but the concentrations of PMMA are different due to the various PMMA: EC ratios utilized to prepare them. Table 2 summarizes the factors and their levels. For the Taguchi design of experiments with three factors and these levels of factors, mixed 2–3 levels from available designs, were chosen and a standard L18 orthogonal array was employed. The S/N ratio has been selected as small is better. Under this condition, the S/N ratio is obtained by Eq. (1).

1 $${\text{S}}/{\text{N}} = - {1}0.{\text{ log}}\left( {\frac{1}{n}{ }\mathop \sum \limits_{i = 1}^{n} Y_{i}^{2} } \right)$$

In this equation, Y_i_ and n stand for the results and number of experiments, respectively. Average capsule size was considered as a response. The confidence level for investigating the significance level using ANOVA was chosen to be 95%. The results were statistically analyzed using Minitab-19 software.

## Results and discussions

### Chemical characterization

**Figure 2:**
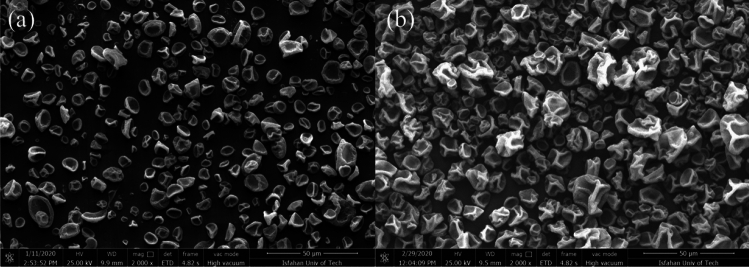
FE-SEM images of the capsules prepared by electrospray process using only a) DCM (S_5_) or b) DMF (S_4_) as solvent. The magnification of the FE-SEM images is $\times$ 2000.

The prepared capsules from various solutions were characterized using FTIR to confirm the presence of EC in the prepared capsules. According to Fig. 1b, the spectrum of neat PMMA exhibits peaks at 1147 cm^−1^ and 1272 cm^−1^, which are associated with O–CH_3_, and C–O stretch in COOCH_3_, respectively. The CH_3_ absorption peaks appear at 1380 cm^−1^, and 1450 cm^−1^. The bands at 1600 and 1730 cm^−1^ prove the existence of the C=O group. The characteristic peaks at 2930 and 2950 cm^−1^ are attributed to the stretching CH in CH_2_^41^. The EC spectrum (Fig. 2b) shows C=O ester, C$\equiv$N, and C=C stretching vibration at 1750 cm^−1^, 2245 cm^−1^, and 1620 cm^−1^, respectively. Moreover, the broad peak of 2870–2990 cm^−1^ and peak at 3470 cm^−1^ represent C–H stretch and O–H vibration, respectively^42^. Figure 1c represents the FTIR spectra of PMMA-based capsules obtained from S_1_, S_2_, S_3_, and S_4_ solutions. Figure 1c confirms the presence of EC in the composition of PMMA-based capsules. This is because all of the spectra represent the characteristic peaks of EC at 2245 cm^−1^ and 1620 cm^−1^, indicating the presence of cyanoacrylate moieties of EC.

### Thermal stability of capsules

The thermal stability of capsules prepared from the S_1_ solution was assessed by TGA under a nitrogen atmosphere. Figure 1d shows the TGA curves of the neat PMMA and EC, along with the capsules containing EC. In Fig. 1d, for the neat PMMA graph, the mass loss starts at 265 °C and leads to a one-stage decomposition at 415 °C which is attributed to the thermal degradation of methyl methacrylate monomer^43^. Figure 1d illustrates the EC thermal decomposition that occurs between the temperature range of 160–380 °C. The TGA diagram of capsules shows the start of thermal decomposition at 175 °C and continues until 435 °C, attributed to the degradation of EC and PMMA, respectively. The thermal decomposition behavior of capsules represents a two-step decomposition. In the first step, there is an approximately 50% weight reduction for capsules before 250 °C with a decomposition rate similar to neat EC. Also, there is a second step for a significant weight reduction after 375 °C with a decomposition rate similar to neat PMMA. The decomposition behavior in the first step is close to the decomposition behavior of neat EC while, in the second step, it is close to the decomposition behavior of PMMA. Therefore, it suggests that the decomposition of the encapsulate (EC) dominates the decomposition behavior of the capsules first while the decomposition of the encapsulant (PMMA) will be significant at higher temperatures, which is in accordance with the thermal decomposition behavior of each species. These results confirm the existence of both EC and PMMA in the prepared capsules^15^. However, for neat EC in comparison to the capsules, there is a slow reduction in weight before 200 °C that can be contributed to EC vaporization while it was prevented when it is capsulated by PMMA. Moreover, the range of decomposition temperature shows the high thermal stability of capsules.

### Effect of parameters on morphology and size

#### Solvent choice and concentration

The effectiveness of electrospray parameters was evaluated by the morphological investigation of the prepared capsules using FE-SEM. Our desired capsules should present a spherical shape and small size without any fibers or holes. DCM and DMF are two kinds of solvents generally used for dissolving PMMA. For choosing the best solvent, pure DCM and DMF were used. According to Fig. 2a, Capsules with pure DCM showed large (micron size), shapeless particles with porous surfaces for all the range of flow rates and applied voltages. Due to the high evaporation rate of DCM, PMMA did not have enough time to rearrange in exerted droplets, and the droplets did not have enough time to change into smaller particles^44^. According to Fig. 2b, Pure DMF caused leaf-like particles with uneven surfaces. These structures were observed for all the range of flow rates and voltages. DMF has a much lower evaporation rate in comparison to DCM, which leads to incomplete evaporation of the solvent, coalescence of particles on the collector, and undesired morphology.

**Figure 3:**
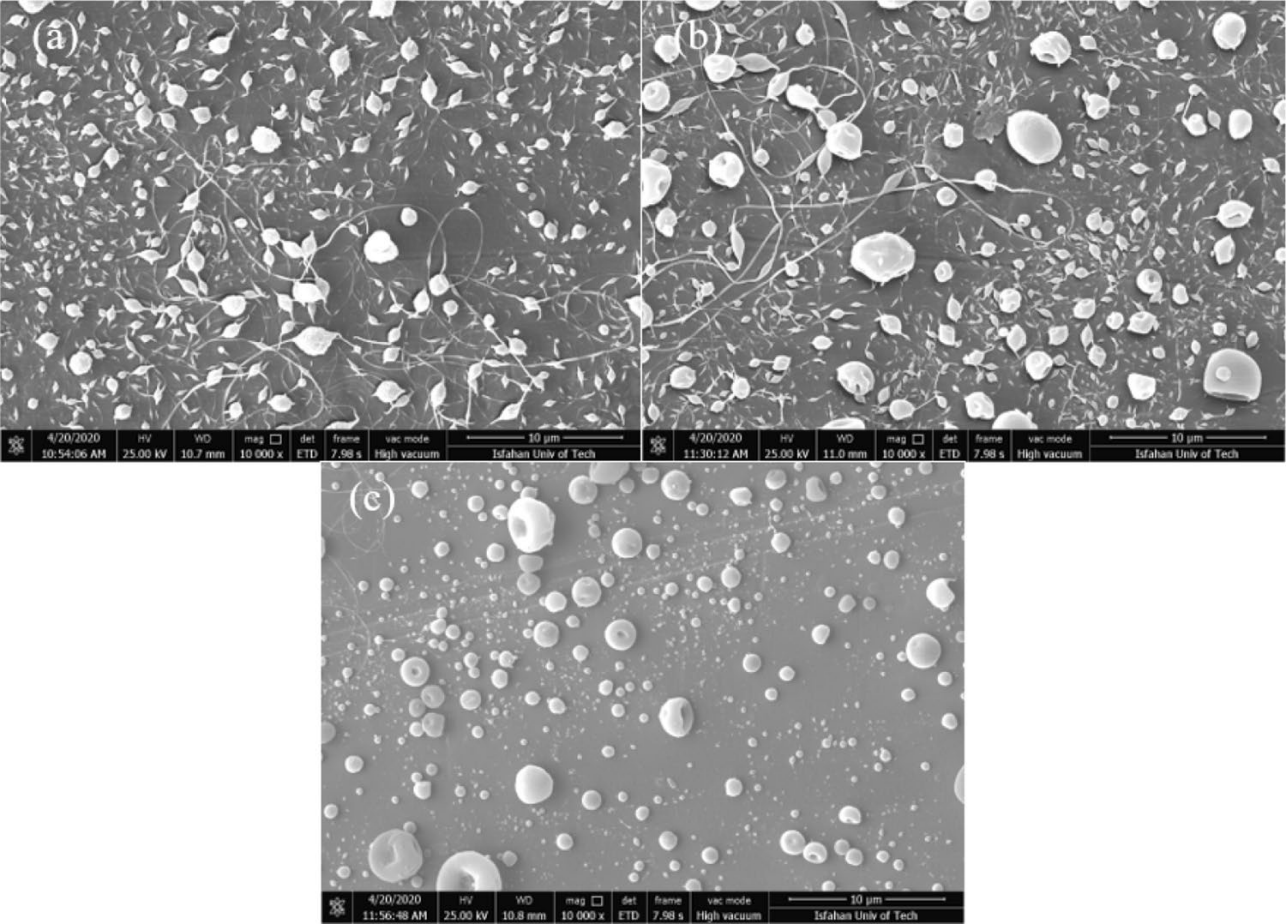
FE-SEM images of capsules prepared by electrospray (**a**) S_2_, (**b**) S_3_, and (**c**) S_1_ solutions. The magnification of the FE-SEM images is $\times$ 10,000.

In order to prepare a solvent with optimized evaporation properties, the same amounts of the solvents were mixed to obtain a 50:50 (v/v) solution of DMF: DCM^22^. At first 60:40 (wt/wt) ratio of PMMA: EC with PMMA concentrations of 3% and 4% was chosen (S_2_ and S_3_ solutions). According to Fig. 3a,b, undesired fibers and irregular-shaped particles were formed in both formulations. While the reduction of PMMA concentration from 4 to 3 wt% reduced the number of particles with undesired morphology, we suppose that the higher concentration of PMMA relative to EC concentration (60:40 wt/wt ratio) keeps the molecular cohesion of the solution high during the electrospray and generally leads to the formation of fibers along with round-shape particles^44^. To evaluate our hypothesis, the concentration of PMMA was reduced to 50:50 (wt/wt) while the overall concentration was kept at 5 wt%. Accordingly, as indicated in Fig. 3c, a significant reduction of undesired fibers and irregular-shaped particles was observed. Based on these results, solutions S_1_ and S_2_ were chosen for further investigation.

#### Flow rate and applied voltage

**Figure 4:**
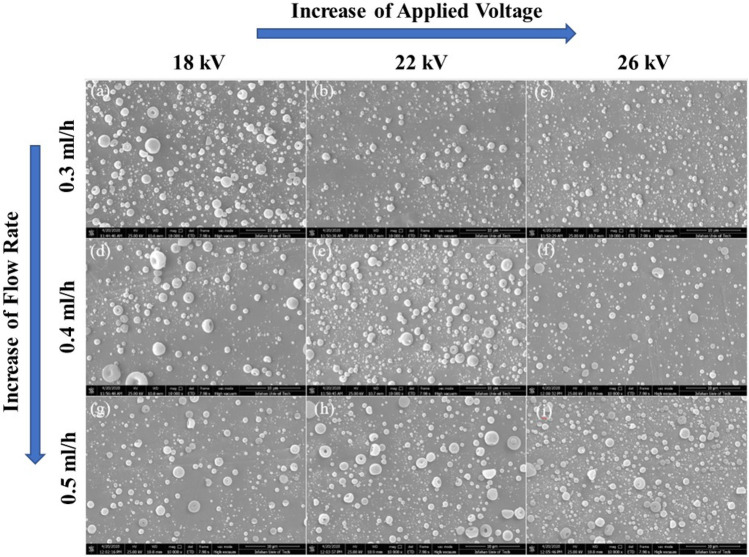
FE-SEM images of the prepared capsules according to the number of experiments provided in Table 3: (**a**) experiment 1, (**b**) experiment 2, (**c**) experiment 3, (**d**) experiment 4, (**e**) experiment 5, (**f**) experiment 6, (**g**) experiment 7, (**h**) experiment 8, (**i**) experiment 9. The magnification of the FE-SEM images is $\times$ 10,000.

**Figure 5:**
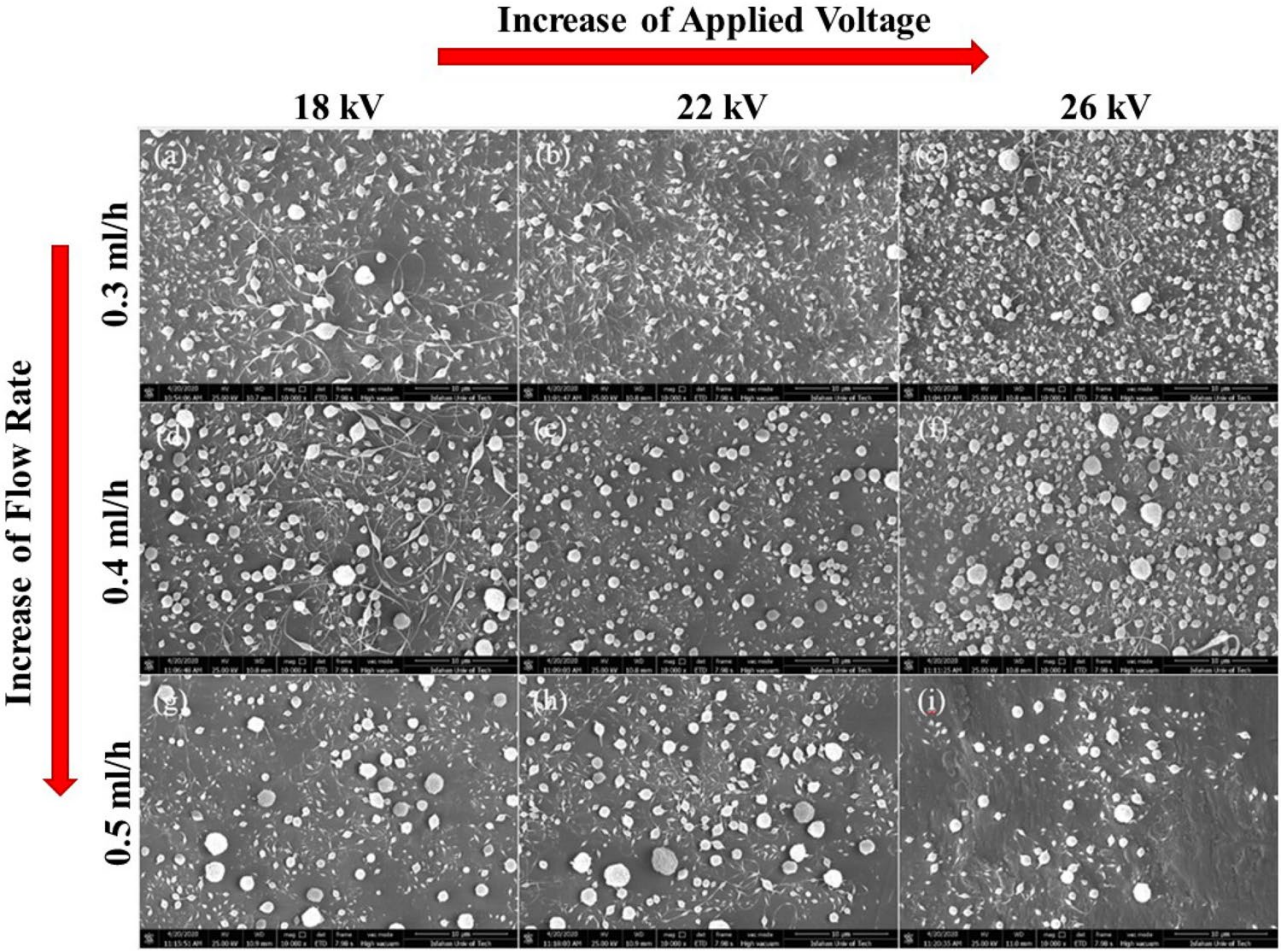
FE-SEM images of the prepared capsules according to the number of experiments provided in Table 3: (**a**) experiment 10, (**b**) experiment 11, (**c**) experiment 12, (**d**) experiment 13, (**e**) experiment 14, (**f**) experiment 15, (**g**) experiment 16, (**h**) experiment 17, (**i**) experiment 18. The magnification of the FE-SEM images is $\times$ 10,000.

The electrospray process of S_1_ and S_2_ solutions (represented as concentrations 1 and 2) was carried out at various flow rates (0.3–0.5 ml/h) and applied voltages (18–26 kV). Table 3 summarizes the parameters and results related to each experiment. Figures 4 and 5 show the FE-SEM micrographs of produced capsules with the specified amount in Table 3. The average diameter of capsules in 18 experiments was obtained by these micrographs and ImageJ software. 50 capsules were chosen for calculating the mean diameter of capsules.

**Table 3:** Results of the electrospray experiments at the specified parameters for S1 and S2 solutions which were capable of producing capsules with intact and spherical morphology.

Experiments	Parameters			Results		
	C^a^	I^b^ (ml/h)*	V^c^ (kV)*	Average diameter of capsules (µm)	Standard Deviation (µm)	S/N ratios
1	1	0.3	18	0.91894	0.4254	0.734257
2	1	0.3	22	0.7069	0.2689	3.01284
3	1	0.3	26	0.67588	0.2485	3.402608
4	1	0.4	18	0.84158	0.4117	1.498092
5	1	0.4	22	0.81914	0.3795	1.732837
6	1	0.4	26	0.74036	0.3003	2.611141
7	1	0.5	18	0.87328	0.3740	1.17693
8	1	0.5	22	0.86208	0.3795	1.289049
9	1	0.5	26	0.842113	0.2884	1.49259
10	2	0.3	18	0.83658	0.2903	1.54985
11	2	0.3	22	0.76386	0.2187	2.339725
12	2	0.3	26	0.7375	0.3000	2.64476
13	2	0.4	18	1.02768	0.3891	− 0.23716
14	2	0.4	22	0.81296	0.2502	1.798616
15	2	0.4	26	0.76478	0.3686	2.32927
16	2	0.5	18	1.10184	0.4365	− 0.84237
17	2	0.5	22	1.07074	0.4578	− 0.59368
18	2	0.5	26	0.89884	0.3463	0.926352

*^a^PMMA/EC concentration ratio, ^b^flow rate, ^c^applied voltage. The mean diameter of capsules and standard deviations were evaluated according to the FESEM micrograph using ImageJ software (n = 50).

**Figure 6:**
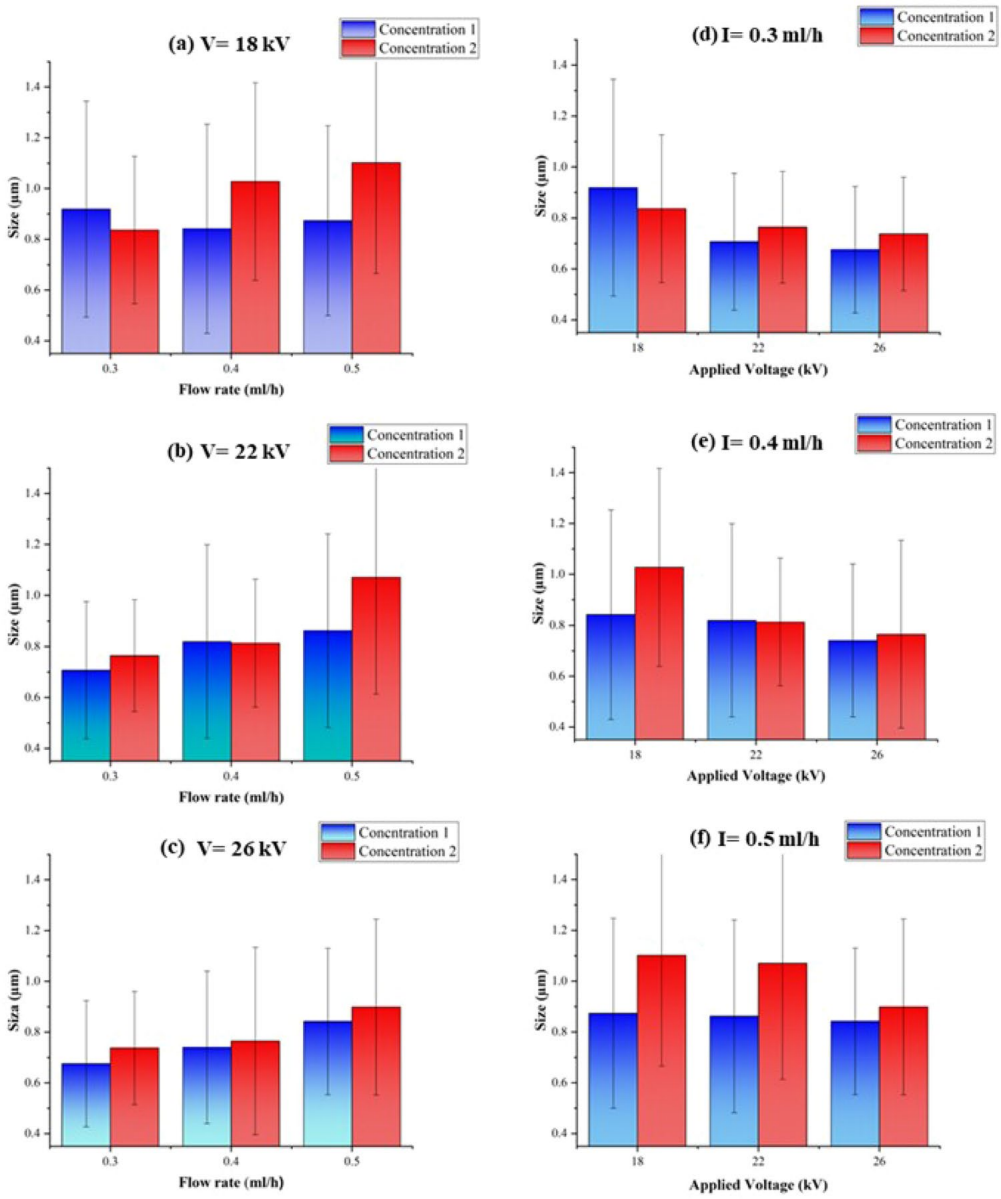
The average size of prepared microcapsules from S1 and S2 solutions at constantly applied voltages of (**a**) 18 kV, (**b**) 22 kV, and (**c**) 26 kV. The average size of prepared microcapsules from S1 and S2 solutions at constant flow rates of (**d**) 0.3 ml/h, (**e**) 0.4 ml/h, and (**f**) 0.5 ml/h. The mean diameter of capsules and standard deviations were evaluated according to the FESEM micrograph using ImageJ software (n = 50).

In order to have general insight, Fig. 6a–c represents the dependence of the average diameter of prepared capsules on the flow rate at constant voltages. Accordingly, the size of prepared capsules slightly increased when the flow rate was increased in a constant applied voltage. In our range of flow rates, the smallest capsules formed at a flow rate of 0.3 ml/h. Our results are in accordance with the previous studies, which showed the increasing flow rate in constant voltage increases the size of capsules because of the disability of the electrical field to atomize droplets^45^.

Similarly, in order to have general insight, Fig. 6d–f represents the dependence of the average diameter of prepared capsules on the applied voltage at constant flow rates. Accordingly, the average capsules’ diameters were slightly reduced by increasing voltage. Moreover, Figs. 4 and 5 show that the applied voltage from 18 to 26 kV at a constant flow rate didn’t affect morphology, and in all FE-SEM images, the capsules with rough and round surfaces were produced. Noteworthy to mention that, to reduce the capsule’s size, an increase in voltage is allowed until the cone jet stays stable. High voltages produce a high electrical field that causes the deformation of the conical meniscus to droplets^45^.

Previously, 2-octyl cyanoacrylate was encapsulated in poly(urethane) by microemulsion method^46^. However, the size of the prepared capsules was as big as 130 μm, and the encapsulation process required the addition of inhibitors in order to prevent the polymerization of cyanacrylate during the encapsulation process as well as using other components such as surfactants. Using these additives will create impurities for such cyanoacrylate-based capsules, which can negatively impact the biocompatibility as well as self-healing efficiency. The electrospray method that was utilized in this work can overcome these challenges by conveniently preparing capsules in the sub-micron size range as well as maintaining the purity of the encapsulate by avoiding using any inhibitors or surfactants.

#### Investigating the effect of processing parameters on the size of capsules using taguchi design

Taguchi design is used for investigating how the size of capsules is changed by effective parameters including the concentration, flow rate, and applied voltage. Eighteen experiments were carried out according to Table 3 and the size of capsules as the results of experiments and S/N ratios are presented in Table 4. These S/N ratios are based on “smaller is better” and are obtained from Eq. (1).

**Table 4:** Analysis of variance for S/N ratios of the size of capsules prepared by electrospray.

Source	DF^d^	Adj SS^e^	Adj MS^f^	F-value	*p*-value
Regression	3	0.18855	0.062851	15.77	0.000
C^a^	1	0.02997	0.029972	7.52	0.016
I^b^	1	0.08488	0.084379	21.30	0.000
V^c^	1	0.07370	0.073700	18.50	0.001
Error	14	0.05579	0.003985		
Total	17	0.24434			

*^a^PMMA/EC concentration ratio, ^b^flow rate, ^c^applied voltage, ^d^degrees of freedom, ^e^adjusted sum of squares, ^f^adjusted mean square.

**Figure 7:**
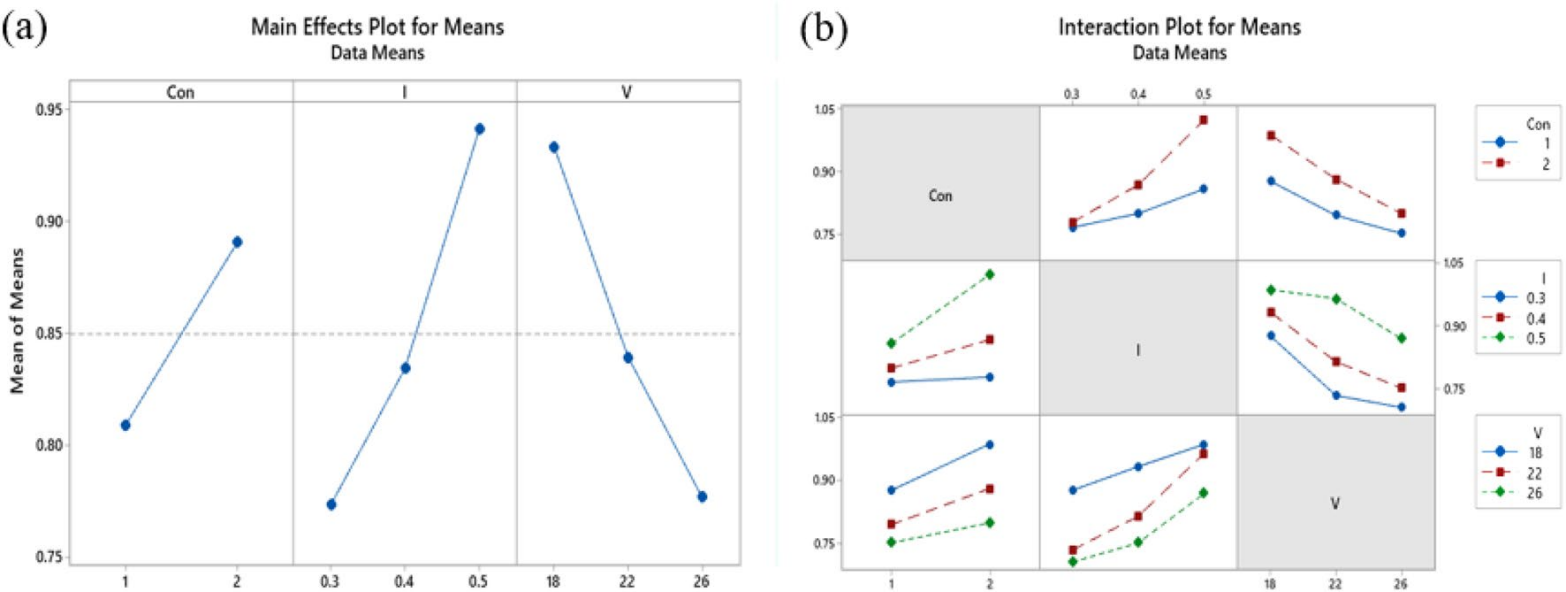
(**a**) Main effects plot and (**b**) Interaction plot for means of the diameter of capsules prepared by electrospray.

Optimal levels of electrospray are obtained in maximum S/N ratios. Large capsules with average diameters of more than 1 µm were achieved in experiments number 13, 16, and 17 with an S/N ratio of − 0.2371, − 0.8423, and − 0.5936, respectively. It can be concluded that negative S/N ratios lead to capsules with a diameter of more than 1 µm. Negative values of S/N were obtained at level 2 of concentration, which is the concentration with a higher amount of PMMA solved in solution (ratio of PMMA: EC equal to 60:40). Consequently, an increase in the concentration of polymers increases the size of capsules. In Fig. 5 fibers can be observed as well. Previous studies showed that decreasing concentration can change the morphology of products from fibers to capsules^47^. As shown in Fig. 4, decreasing concentration resulted in the formation of capsules without fibers. Table 4 shows the results of ANOVA from regression analysis. The parameter with a 0.000 P-value has the highest prominence. Due to the 95% significance level, P-values less than 0.05 are significant. So, flow rate, voltage, and concentration with P-values 0.000, 0.001, and 0.016, respectively, are significant parameters. There is also information on the degree of freedom (DF), the adjusted sum of squares (Adj SS), the adjusted mean of squares (Adj MS), and F-values in Table 4. These parameters are used for the investigation of the P-value. According to the Mean of Means plot in Fig. 7, for achieving the minimum size of capsules, concentration, and flow rate should be at their lower levels and voltage should be at the higher level (in specified levels). Also, the amount of S/N ratios is in good agreement with the Mean of Means plot.

**Figure 8:**
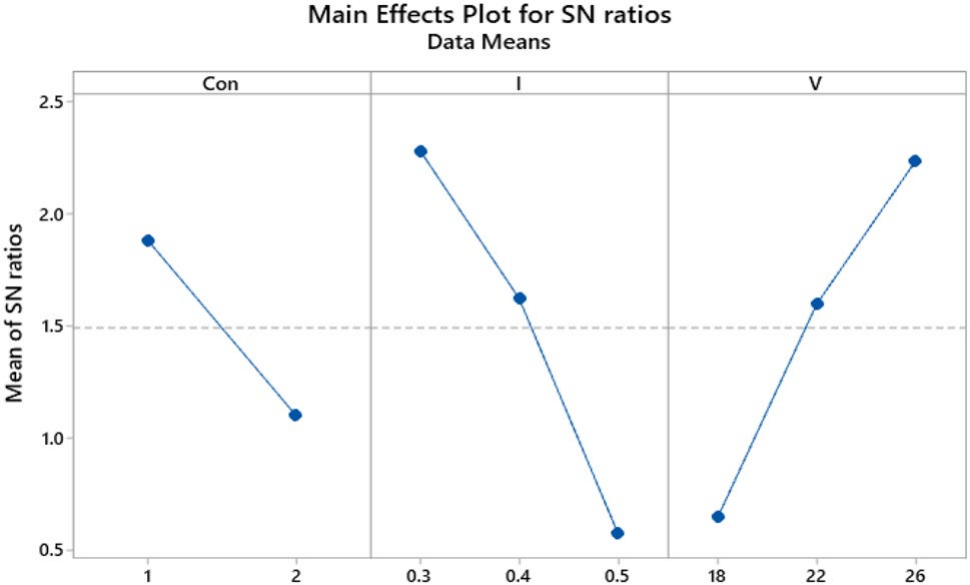
Main effects plot for S/N ratios of the diameter of capsules prepared by electrospray.

The S/N ratio plot for the mean diameter of the capsules is shown in Fig. 8. Also, Table 5 shows the S/N ratio between the maximum and minimum main effects. According to Taguchi design, the highest difference between S/N ratios is the most significant value. The most effective factor is flow rate followed by voltage and polymer concentration with 1.7, 1.56, and 0.78 values, respectively.

**Table 5:** Response table for S/N ratios.

Level	C^a^	I^b^	V^c^
1	1.8834	2.2807	0.6466
2	1.1017	1.6221	1.5966
3		0.5748	2.2345
Delta	0.7817	1.7059	1.5879
Rank	3	1	2

*^a^PMMA/EC concentration ratio, ^b^flow rate, ^c^applied voltage.

## Conclusion

In this research, the electrospray method was used for the encapsulation of EC in PMMA capsules. Chemical characterization of capsules via FTIR tests confirmed the presence of both core and shell material without any reaction. TGA results revealed the thermal stability of capsules at more than 175 °C. FE-SEM micrographs indicated the morphology of capsules from unshaped objects and fibers to capsules, which were modified by changing electrospray parameters. Utilizing various flow rates (0.3–0.5 ml/h) and applied voltage (18–26 kV), capsules were obtained with a 0.6–1 μm size range. Therefore, investigations were performed into the production of the small capsules. Taguchi design was used for assessing the effect of three electrospray parameters including flow rate, voltage, and solution concentration on the size of capsules. At constantly applied voltages, the increment of flow rate increased the capsule size up to 40% (ANOVA, *p* ≤ 0.05), while at constant flow rates, the increase in applied voltage reduced the average capsule size by 3.4–26% (ANOVA, *p* ≤ 0.05). In the current study, the optimum size of capsules was the smaller ones which could be obtained at 2.5 wt% of PMMA as encapsulant concentration, 0.3 ml/h flowrate, 26 kV applied voltage, and 50:50 (wt/wt) PMMA to EC ratio as the encapsulant to encapsulate ratio. This research showed a convenient fabrication method of micron-sized cyanoacrylate bioadhesive by electrospray that can be possibly used for self-healing biomedical applications, although it requires further investigation.

## Data Availability

The datasets used and/or analysed during the current study are available from the corresponding author on reasonable request.
